# The proteins of intra-nuclear bodies: a data-driven analysis of sequence, interaction and expression

**DOI:** 10.1186/1752-0509-4-44

**Published:** 2010-04-13

**Authors:** Nurul Mohamad, Mikael Bodén

**Affiliations:** 1 Institute for Molecular Bioscience, The University of Queensland QLD 4072, Australia

## Abstract

**Background:**

Cajal bodies, nucleoli, PML nuclear bodies, and nuclear speckles are morpohologically distinct intra-nuclear structures that dynamically respond to cellular cues. Such nuclear bodies are hypothesized to play important regulatory roles, e.g. by sequestering and releasing transcription factors in a timely manner. While the nucleolus and nuclear speckles have received more attention experimentally, the PML nuclear body and the Cajal body are still incompletely characterized in terms of their roles and protein complement.

**Results:**

By collating recent experimentally verified data, we find that almost 1000 proteins in the mouse nuclear proteome are known to associate with one or more of the nuclear bodies. Their gene ontology terms highlight their regulatory roles: splicing is confirmed to be a core activity of speckles and PML nuclear bodies house a range of proteins involved in DNA repair. We train support-vector machines to show that nuclear proteins contain discriminative sequence features that can be used to identify their intra-nuclear body associations. Prediction accuracy is highest for nucleoli and nuclear speckles. The trained models are also used to estimate the full protein complement of each nuclear body. Protein interactions are found primarily to link proteins in the nuclear speckles with proteins from other compartments. Cell cycle expression data provide support for increased activity in nucleoli, nuclear speckles and PML nuclear bodies especially during S and G_2 _phases.

**Conclusions:**

The large-scale analysis of the mouse nuclear proteome sheds light on the *functional *organization of *physically *embodied intra-nuclear compartments. We observe partial support for the hypothesis that the physical organization of the nucleus mirrors functional modularity. However, we are unable to unambiguously identify proteins' intra-nuclear destination, suggesting that critical drivers behind of intra-nuclear translocation are yet to be identified.

## Background

The nucleus not only houses the genetic material but also administers its transcription. Morphologically defined intra-nuclear compartments or bodies are non-randomly interspersed amongst chromosomes. Preliminary evidence indicates that these compartments are spatially and functionally organized, and play important regulatory roles. For example, nucleoli are primarily concerned with ribosomal biogenesis at sites of ribosomal genes, nuclear speckles accumulate pre-mRNA splicing factors at actively transcribed genes, and nuclear pore complexes sit in the nuclear membrane to control import and export of material. Other compartments, such as Cajal and PML nuclear bodies, appear to be involved in a much broader range of functions.

The modular nature of the nuclear milieu and the fluid means of molecular transport enable several levels of regulation. For example, PML nuclear bodies assemble in response to sumoylation (a post translational modification; [1]). They also retain and release regulatory proteins in response to cellular stress (e.g. p53; [2]). Similarly, the nucleolus sequesters regulatory proteins for timely release (e.g. Mdm2; [3]). At a more general level, nuclear pores regulate protein access to the nucleus [4].

This paper paves the way for the computational mapping of the role of nuclear architecture in critical regulatory processes. It taps recent experimental data and the growing knowledge of nuclear architecture to portray the function and the protein complement of intra-nuclear compartments. It is important to note that proteins and RNA do not constitutively and exclusively localize to a single nuclear compartment. Data sets and models need to account for this biochemically fluid and dynamic organization. For instance, several proteins associate transiently with nuclear bodies under stress conditions [5]. We perform analyses that help shed light on the character of compartments in terms of their proteins: known functional features, interactions and expression profiles. Moreover, we develop predictive models that are capable of predicting compartment associations of any amino acid sequence that avails itself to the nuclear environment.

We hypothesize that the high level of physical organization can be explained by advantages brought by functional modularity. We expect that large-scale data will reflect such functional relations (e.g. that compartment members are functionally homogeneous) as well as features that enable and implement this organization (e.g. that compartment members carry signals that assist their intra-nuclear translocation). Using the *M. musculus *nuclear proteome as a reference we assemble experimental data about intra-nuclear compartments. This reference set enables us to take a system's view on a range of different properties. Firstly, we identify the compartment-specific enrichment of gene ontology terms to uncover the properties shared amongst and specific to members. Secondly, we investigate what sequence properties can be used to characterise or even identify the compartment with which a protein associates. We build compartment-specific classifiers based on support-vector machines (SVMs) to capture information in amino acid sequence data. Thirdly, we cross-reference intra-nuclear compartment and protein-protein interaction data to evaluate the extent compartments are linked via their members. Similarly, mRNA levels collected over the cell cycle are used to investigate the temporal expression profile of members of each compartment. The study focuses specifically on the dynamic nuclear bodies; Cajal bodies, PML nuclear bodies, and nuclear speckles [6]. We include the nucleolus that, similar to the aforementioned bodies, changes its morphology over the cell cycle. Compartments with a more static appearance, such as the nuclear pore complex, chromatin and nuclear envelope [7] are, for purposes of this paper, grouped and labelled 'Other'.

## Results

The association of proteins to intra-nuclear bodies and documented nuclear import are gleaned from multiple resources. Specifically, we use the Nuclear Protein Database (NPD) [7] and generic databases including UniProtKB [8] and the Human Protein Reference Database (HPRD) [9]. We further identify proteins from the recent literature as detailed in Methods. We intersect the compartment-annotated data with the mouse nuclear proteome (Nucprot) [10]. When applicable, we map annotations for human proteins to mouse via orthology. The final set consists of 2603 proteins with 1293 proteins with one or more experimentally confirmed intra-nuclear compartments (of which 956 associate with one or more of the nuclear bodies listed above). The remaining 1310 proteins are only known to localize generally to the nucleus.

The nucleoli is the largest compartment both physically and in terms of proteins. Of the 2603 nuclear proteins in our reference set, 578 associate with the nucleoli. 392 associate with the nuclear speckles, 83 with PML nuclear bodies, and only 36 with Cajal bodies. These numbers reflect the variety of proteins that truly associate with each compartment, and compartment size, but also experimental biases (e.g. the difficulty of biochemically isolating them and thus discovering their protein complement). It is important to note that this sampling bias may influence the results below to a smaller degree.

Proteins are not exclusively associated with one compartment and this is evident in our analysis (see Table 1). In fact, many proteins shuttle between them, in particular the nucleolus and nuclear speckles. (Note that Table 1 only counts proteins that are annotated with a single or exactly two locations.)

**Table 1:** Proteins annotated with exactly one and only two compartments.

Intra-nuclear body	Nucleolus	Nuclear speckle	PML nuclear body	Cajal body	Other
Nucleolus	**379**				
Nuclear speckle	71	**253**			
PML nuclear body	5	3	**44**		
Cajal body	5	8	0	**14**	
Other	67	25	18	2	**256**

The number of different proteins belonging to a single body are shown in the diagonal. The number of different proteins that occur in exactly two bodies are shown in the off-diagonal cells. The row and column headers identify the bodies that share the protein. Nuclear proteins that belong to zero or two or more compartments are excluded.

### Analysis by annotation

In the following, we make observations of functions and biological processes of proteins that co-localize according to their annotations. Specifically, we evaluate the statistical enrichment of Gene Ontology (GO) terms of proteins belonging to the same nuclear body (as opposed to belonging to any other body in the nucleus; more details about the tests and their interpretation are found in Methods). GO terms are expert-curated descriptors for genes and their products. They fall into three distinct taxonomies: biological process, molecular function, and cellular component.

In Table 2, the statistically enriched GO terms are shown at a significance level *E *< 0.01. (That is, of those listed we expect to see less than 0.01 terms identified by chance alone.) The nucleoli are central to ribosome formation [11]. Much of the ribosome assembly, such as rRNA transcription, occurs within the nucleoli. In fact, previous bioinformatics analyses of proteomic data have classified nucleolar proteins into several functional groups. Approximately 30% of proteins have a function related to the production of ribosome subunits [12]. The present analysis re-affirms this core activity of nucleolar proteins. Recent data have also demonstrated a range of other cellular functions of nucleoli relating to the inheritance of genetic disorders, predisposition to cancer as well as cellular senescence and stress response [12]. In our set, the group of proteins that exhibit such terms within the nucleolus is not significantly over-represented.

**Table 2:** GO Terms assignment for intra-nuclear bodies.

Intra-nuclear body	GO Term	Description	E-Value
**Nucleolus**	GO:0042254	Ribosome biogenesis and assembly	1.80E-19
	GO:0022613	Ribonucleoprotein complex biogenesis and assembly	3.30E-15
	GO:0016072	rRNA metabolic process	3.30E-14
	GO:0006364	rRNA processing	3.30E-14
	GO:0009059	Macromolecule biosynthetic process	2.90E-08
	GO:0043228	Non-membrane-bound organelle	3.30E-08
	GO:0043232	Intracellular non-membrane-bound organelle	3.30E-08
	GO:0006412	Translation	5.70E-08
	GO:0009058	Biosynthetic process	1.20E-06

**PML nuclear body**	GO:0006950	Response to stress	6.10E-09
	GO:0006974	Response to DNA damage stimulus	1.70E-08
	GO:0009719	Response to endogenous stimulus	2.30E-08
	GO:0050896	Response to stimulus	4.00E-07
	GO:0006281	DNA repair	4.40E-05
	GO:0042770	DNA damage response, signal transduction	5.90E-05
	GO:0007049	Cell cycle	1.40E-03
	GO:0006464	Protein modification process	1.60E-03
	GO:0022402	Cell cycle process	2.70E-03

**Nuclear Speckle**	GO:0016071	mRNA metabolic process	1.20E-61
	GO:0006397	mRNA processing	4.80E-61
	GO:0008380	RNA splicing	1.20E-58
	GO:0005681	Spliceosome	1.90E-38
	GO:0006396	RNA processing	5.80E-34
	GO:0030529	Ribonucleoprotein complex	1.90E-26
	GO:0003723	RNA binding	2.00E-25
	GO:0016070	RNA metabolic process	1.70E-06
	GO:0000377	RNA splicing, via transesterification reactions with bulged adenosine as nucleophile	4.10E-06
	GO:0000398	Nuclear mRNA splicing, via spliceosome	4.10E-06

For each compartment, GO terms assigned to member proteins that are also deemed significantly enriched (relative the terms assigned to the pool of nuclear proteins) are shown using the term ID and the description. The *E*-value is the Bonferroni corrected *p*-value of the term as determined by the Fisher's exact test (see Methods). The *E*-value is below 0.01 for all terms shown. GO terms for the nuclear compartments and their parts are excluded.

Other compartments have a similar functional repertoire. Indeed, and as confirmed by the GO term enrichment analysis, proteins that localize to PML nuclear bodies are involved in a variety of nuclear processes including DNA damage and stress response, apoptosis and cell cycle regulation [13,14]. Proteins that localize to PML nuclear bodies, partially or temporarily, are functionally diverse and the enrichment analysis identifies a few roles. Tumour suppressor proteins including Tp53, Cbp and Hipk2 are involved in post-translational modification processes and contribute to crucial functions of PML nuclear bodies. For instance, phosphorylation by Hipk2 and acetylation by Cbp contribute to sensing cellular stress signals and the response to DNA damage.

Nuclear speckles are enriched in pre-mRNA splicing factors. In addition, nuclear speckles also house several kinases and phosphatases that phosphorylate and dephosporylate components of the splicing machinery [15]. The overall role of splicing speckles aligns with the regulation of splicing factors' access to the splicing machinery (Table 2).

The GO term with the strongest enrichment for Cajal bodies is 'spliceosome assembly' at *E *< 1 (not shown in Table 2). The weak enrichment is probably caused by the small and incomplete set of proteins known to localize to these bodies. We thus make a few anecdotal remarks. There is growing evidence implicating Cajal bodies with snRNP and snoRNP biogenesis [16]. The presence of Tp53 in Cajal bodies during stress suggests the potential involvement of this compartment in the cellular response to stress [16]. That Cajal bodies contain several nucleolar proteins (including fibrillarin) suggest that they are involved in preribosomal RNA processing [17]. Additionally, Cajal bodies are implicated in histone pre-mRNA 3' maturation and basal transcription factor of RNA polymerases I, II and III and telomerase RNA [18,19].

### Analysis by sequence

This section explores pre-defined sequence patterns relating to intra-nuclear organization, potentially specific to compartments, namely nuclear localization signals and sumoylation sites. This section also investigates more subtle sequence features as compartment determinants by training classifiers that are evaluated (on held-out proteins for which compartments are known) and then used to characterize proteins with no presently known nuclear compartment.

Nuclear localization signals (NLSs) are short amino acid sequences that induce nuclear import by binding to so-called Importins. NLS-based import is believed to be the most common (and best understood) mechanism for proteins to enter the nucleus but not the only one. To explore the effect of NLS on intra-nuclear sorting, we identify NLS-carrying nuclear proteins by matching the amino acid sequence to the 312 motifs in NLSdb [20]. Bickmore and Surtherland [21] report that by using PSORT they detect NLS in 80-86% proteins that localize to nucleoli, nuclear speckles and PML nuclear bodies; and 62% of proteins that associate with Cajal bodies. NLSdb is more strict and a smaller number of positive predictions are expected generally.

Only nuclear speckles show a larger proportion of NLSs than by chance alone (*p *< 0.05 using a Fisher's exact test; relative the compartment-annotated nuclear proteome). At 38%, NLS-containing proteins are more common in speckles than in other nuclear bodies. Other bodies have 29-34% NLS-containing proteins. Sumoylation is a post-translational modification of proteins, attaching a small ubiquitin-like modifier protein (SUMO) to a lysine in the substrate. Evidence suggests that sumoylation plays a role in preserving function and integrity of intra-nuclear compartments [22]. For instance, many of the proteins localize to PML nuclear bodies are reported to be sumoylated [14], and SUMOylation has been suggested to be responsible for the assembly of these bodies [1]. For instance, sumoylation is necessary for the recruitment of PML nuclear bodies components such as SP100.

To explore the role of sumoylation in the organization of compartments, proteins that contain a putative sumoylation site are identified.

The sumoylation consensus motif is defined as Ψ-K-X-E (where Ψ is a hydrophobic amino acid, K is a Lysine and E is Glutamic acid). This motif is used to scan all proteins, and all proteins with at least one hit are considered positives. Overall, 41% of nuclear proteins have this motif. We employ a statistical test to observe which bodies that are significantly enriched for the sumoylation motif (*p *< 0.05 using the Fisher's exact test; relative the compartment-annotated nuclear proteome). The analysis shows that sumoylation predominantly occurs in PML nuclear bodies (with 61% sumoylation motif-containing proteins). The other compartments are not significantly enriched with sumoylated proteins (with 31%, 41%, and 38% for Cajal body, nuclear speckle, and nucleolus, respectively).

The existence of compartment-specific sequence features is further evaluated using SVM machine learning classifiers. SVMs have been successfully applied to discover and leverage sequence similarities not easily represented as linear motifs to build predictive models. To investigate if there are compartment-specific determinants in the amino acid sequences, we build classifiers from the available data and evaluate their ability to generalise to unseen proteins. Classifiers with high accuracy can be used to understand yet unclassified sequences, e.g. nuclear proteins with unknown intra-nuclear associations.

The development of the prediction methods for intra-nuclear proteins is exemplified by work by Lei and Dai [23]. They predict a single intra-nuclear localization using SVMs. Multi-localized proteins are excluded and thus their model ignores the large and important fraction of proteins that transiently associate with several compartments. Lei and Dai report an accuracy as measured by the correlation between the prediction and the actual compartment ranging from 0.26 to 0.42 (1 is perfect, 0 is random).

We include and handle multi-localized proteins. Specifically, we cast compartment prediction as multiple binary classification problems, where proteins of one nuclear body are labeled as positives and all proteins from other compartments are negatives (see Additional file 1 for protein identifiers and compartments when available). Different configurations are tested and we report only on the best below (see Methods for details). We set aside the set of proteins that have no intra-nuclear location assigned. (This set is used later to identify novel candidates for each of the compartment that we build classifiers for.)

To illustrate the overall performance of classification on basis of sequence data, we generate Receiver Operating Characteristic (ROC) curves for the SVMs equipped with the Local Alignment kernel [24]. The ROC curve illustrates the rate of true positives as a function of the rate of false positives (when testing different thresholds on the raw SVM output; see Methods for output function).

**Figure 1:**
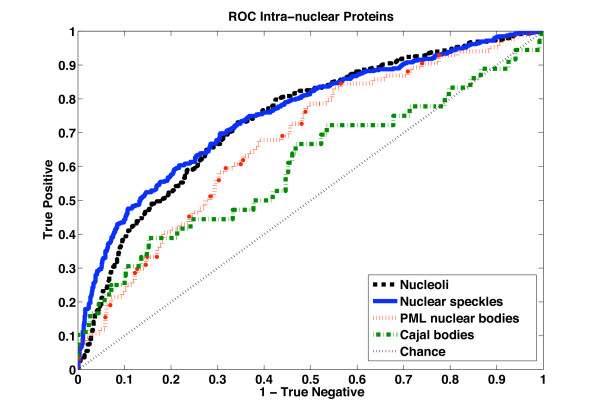
**ROC curves of intra-nuclear proteins**. The ROC curves illustrate the accuracy of detecting Nucleolar, Cajal body, Nuclear speckle and PML body proteins over all classification thresholds. Chance prediction accuracy is illustrated by the diagonal.

In Figure 1, we note that the classification accuracy is high for nucleoli and nuclear speckles. Slightly lower performance is achieved for the functionally heterogeneous PML nuclear bodies. Cajal bodies proteins are classified poorly. The positive sets are very small for the latter two nuclear bodies (83 and 36, respectively), a property that is likely to adversely affect classification accuracy. We note the area under the ROC curve (AUC) in Table 3 determined for held-out data (via cross-validation) for each body. An AUC of 0.75 for nuclear speckles and 0.73 for nucleolar proteins indicate relatively high accuracy (an AUC of 1.0 is perfect classification and 0.5 is chance performance) and that distinguishing sequence features exist.

**Table 3:** Compartment-specific classifiers mean and stand deviations

Intra-nuclear body	Mean	Standard Deviation
Nucleolus	0.734	0.006
Nuclear speckle	0.757	0.004
PML nuclear body	0.670	0.021
Cajal body	0.583	0.032

The mean accuracies and standard deviations for compartment-specific classifiers when tested on data with-held during training. All classifiers use the Local Alignment Kernel and tests are repeated ten times with different data set divisions.

Each classifier is next used to screen the 1310 protein set with no known intra-nuclear location. To provide a reasonable estimate of total numbers of compartment members, we first determine a suitable threshold. Specifically, we set the SVM's threshold such that approximately the same number of positives would have been generated on the data for which intra-nuclear compartments are known. The thresholding of the un-annotated novel set results in 287 nucleoli proteins, 309 nuclear speckles proteins and 52 PML nuclear bodies proteins and 55 Cajal bodies proteins of the 1310 un-annotated protein set (see Additional file 2 for all predictions). Gleaning the annotated data, we calculate the expected False Discovery Rate (FDR) for each compartment. For nucleoli and nuclear speckles, the FDR is 0.36 and 0.43, respectively. Hence, of the 300 or so predicted positives, about 40% are expected to be incorrect. In contrast, the PML nuclear bodies and Cajal bodies predictors have an FDR of 0.84 and 0.86, respectively. It is important to note that the negative set is much larger than the positive set for each of these compartments, naturally leading to many false discoveries. We also expect there to be a large number of proteins in the training set that due to the lack of experimental data are wrongly labelled as negatives.

Sequence features that are found by predictors potentially correspond to functional properties of proteins. We are therefore interested in seeing to what extent such features are shared between proteins of different compartments. From the accuracy of prediction we already know that nucleolar and nuclear speckle proteins are the easiest to predict. More detail is found in Table 4 that shows the number of proteins that are predicted by each compartment-specific predictor. Specifically, by using the same thresholds as we set above, we determine for each predictor, how many predicted proteins that are in fact false positives, and belong to another compartment. It appears that a high proportion of Cajal body members are positively identified by the nucleolus predictor (12/36 = 33% of all Cajal body proteins) indicating that nucleolar proteins may share features with the small set of Cajal body proteins. Indeed, some Cajal body components including fibrillarin are located in Cajal body before accumulating in nucleoli [16]. In neurons, approximately 30% of Cajal body proteins are attached to the nucleolus, suggesting that the association may play an important role in delivering snoRNPs to the nucleolus [25]. Similarly, 24/83 = 29% of all PML body proteins are predicted by the nucleolus predictor. We also note that the PML body predictor identifies 22/392 = 6% of all nuclear speckle proteins, and that the nuclear speckle predictor finds 20% of all PML body proteins and 20% of all Cajal body proteins. It is interesting to note that PML bodies are often physically juxtaposed with both nuclear speckles and Cajal bodies [13]. Short actin- or lamin-based fibres have been suggested to transiently link such domains [26]. Overall, we are unable to fully explain the occurrence of false positives, but there appears to be biologically meaningful circumstances for the confusion.

**Table 4:** Number of proteins predicted by compartment-specific predictor.

Predictor	Actual compartment	Predictions
Nucleolus vs other (578)	Nucleolus	**373**
	Nuclear speckle	78
	PML nuclear body	24
	Cajal body	12

Nuclear speckles vs other (392)	Nucleolus	94
	Nuclear speckles	**223**
	PML nuclear body	16
	Cajal body	7

PML nuclear body vs other (83)	Nucleolus	18
	Nuclear speckle	22
	PML nuclear body	**12**
	Cajal body	1

Cajal body vs other (36)	Nucleolus	17
	Nuclear speckle	6
	PML nuclear body	2
	Cajal Body	**5**

For each compartment-specific predictor, the known (experimentally determined) compartment is shown for all "positive" predictions (total number of positive predictions are shown in parenthesis). The last column shows the number of predictions that knowingly belong to the compartment identified in the second column. For example, the test outcomes of the predictor trained to discriminate nucleolar proteins from others are shown in the first section. Of 578 positives, 373 were correctly classified, 78 were classified as "nuclear speckle" proteins, etc. Note that in the case of multi-class proteins, the alternative compartment is not necessarily incorrect.

### Analysis by protein-protein interaction data

It is widely held that proteins associate with intra-nuclear compartments via molecular interaction. However, interaction is often transient and proteins move between compartments by anomalous diffusion [27]. We are interested in understanding if there is a pattern of "interaction exchange" between compartments reflected by current data collections. This section inspects the interactions of individual proteins, given their association with intra-nuclear compartments. In particular, we ask, to what extent is there pairwise interactions between members of different compartments?

We consider the extensive protein-protein interaction data from HPRD [9] and map this data to mouse proteins by orthology (see Methods for details). To simplify the analysis, we include only interactions between proteins that are each annotated with a single nuclear body or compartment. This means that each interaction is strictly either intra- or inter-compartmental.

Table 5 shows the proportion of protein interactions that take place within the same body versus the proportion of protein interactions between nuclear bodies. Proteins that belong to any other compartment (not a nuclear body) are categorized as 'Other'.

**Table 5:** Protein interactions between intra-nuclear bodies.

Intra-nuclear body	Nucleolus (159)	Nuclear speckle (93)	PML nuclear body (38)	Cajal body (10)	Other (479)
Nucleolus	**54**				
Nuclear speckle	13	**34**			
PML nuclear body	5	3	**11**		
Cajal body	0	1	0	**2**	
Other	87	42	19	7	**324**

The number of intra-compartmental interactions are shown in the diagonal. The number of inter-compartmental interactions are shown in the off-diagonal cells (that is, all interactions involving proteins associated with more than body are excluded; see Methods).

Intra-compartmental interaction is typically far more frequent than any single inter-compartmental interaction. (Interactions involving the sparsely populated Cajal bodies and the non-distinct 'Other' category are exceptions.) This general observation supports that protein-protein interaction is a primary force behind intra-nuclear organization. Due to the small number of samples, specific observations involving two different locations are not supported statistically.

Our analysis only concerns nuclear imported proteins, but we note that the protein interaction data can contain sampling biases [28] at a fine-grained, "compartment" level. We inspected the supporting literature for a subset (10) of the interaction entries and in no case did the experiment design explicitly involve intra-nuclear compartments. We believe that the risk is low that interaction profiles for compartments are artificially skewed.

### Analysis by cell cycle expression

The cell cycle is a major and critical biological process in the cell, representing the stages of replication (S) to cell division (M). Understanding how intra-nuclear bodies relate to the stages uncovers possible links to other cell cycle components and elucidates the compartments' regulatory roles. During the cell cycle, most intra-nuclear bodies change profoundly in terms of their biochemical composition. Assembly and disassembly of some compartments appear to correlate with cell cycle stage (e.g. PML nuclear bodies; [1]). Some genes are only expressed at a specific stage of the cell cycle, allowing us to investigate if proteins for a compartment tend to avail themselves selectively during the cell cycle. Specifically, we look closer at the proportion of proteins associated with each compartment that are periodically expressed in the cell cycle. Cyclebase contains the expression of human genes as measured during the cell cycle [29]. It provides for each gene an assessment whether it is differentially expressed and the periodicity of such expression (represented by a specific cell cycle "peaktime"). First, human genes are mapped to their mouse orthologs. We then extracted the peaktime of expression for each gene, incorporating interphase (G_1_, S, G_2_) and mitosis (M). (Genes with uncertain peaktime are excluded from the analysis.) In total, 817 genes (coding for our mouse nuclear proteome) have a peaktime assigned. Out of this set, 21 and 54 genes code for nuclear speckle and nucleolar proteins, respectively. For PML nuclear bodies and Cajal bodies, we find only 10 and 3 genes with a peaktime assigned, respectively. The analysis below must thus be interpreted in light of these limited numbers.

**Figure 2:**
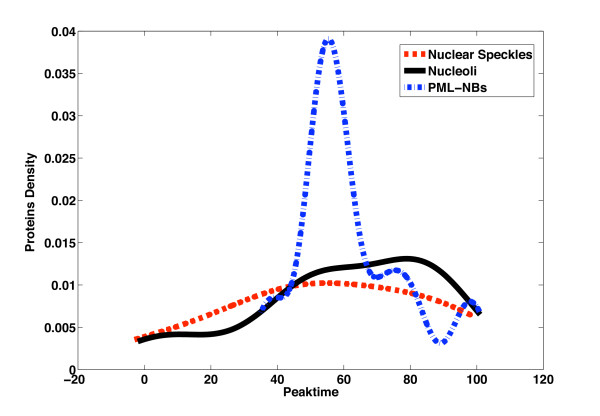
**The distribution of the compartment-specific proteins over the cell cycle**. The plot shows the distribution over the cell cycle (represented by expression peaktime) of compartment-specific proteins, coded for by periodically expressed genes. In total, 54 nucleolar genes, 21 nuclear speckle genes and 10 PML nuclear body genes are shown. The distribution is determined by kernel density estimation (MATLAB 'ksdensity', default settings).

Figure 2 shows the proportion of "periodic" proteins that are expressed over the cell cycle. The peaktime is given as percentage of a cell cycle and it refers to a measure of when the gene is maximally expressed [30]. The transition of M/G_1 _is represented by peaktimes 0 and 100. S and G_2 _correspond to peaktimes 47-70% and 71-89%, respectively. Kernel density estimation is used to visualize the peaktime of nucleolar, nuclear speckle and PML nuclear body proteins (the periodic Cajal body proteins are too few to be shown). We make specific and anecdotal observations for each compartment below to corroborate the role of periodic expression in each.

#### Nucleoli

Nucleoli are dynamic structures that assemble in interphase during transcription. As seen in Figure 2, nucleoli-associated periodic genes are indeed expressed in interphase with a slightly bi-modal distribution (at S and G_2_; containing approximately 40% and 30% of 54 genes, respectively). As an example, Cdk7 and Cdk9 (members of the cyclin-dependent kinases) are expressed at this time, modifying and thereby controlling other proteins. Cdk7 is also expressed during mitosis.

Nucleoli also contain proteins controlling DNA replication. Some of the responsible genes are selectively expressed in S phase are members of the minichromosome maintenance (MCM) protein family (including Mcm2, Mcm3, Mcm4, Mcm5 and Mcm6). Mcm4 is also observed to express in M phase suggesting a role beyond replication.

Nucleolus stress response is exemplified by the periodic expression of Cdkn2a in S phase. Cdkn2a interacts with and sequesters Mdm2 and P19arf proteins to indirectly activate tumor suppressor protein Tp53 [12]. (Mdm2 normally degrades Tp53 by ubiquitination. However, stress stimuli increases the expression of P19arf that associates with Mdm2 and blocks the ubiquitination and subsequent degradation of Tp53.)

#### Nuclear speckles

Nuclear speckles recruit many transcription and splicing factors. Only 21 of 392 nuclear speckle proteins have genes that are periodically expressed. 57% and 34% of these 21 genes are expressed in G_1 _and S phase, respectively. 5 genes are expressed in G2 and 5 in M phase (23% each). Any clear pattern is therefore not seen in Figure 2. Anecdotally, we note that at M/G_1_, Nxf1 and Hspa8 are expressed. Nxf1 is a gene that is involven in the export of mRNA from nucleolus to cytoplasm. Hspa8 is a member of the heat shock protein family and functions as a molecular chaperone for newly synthesized proteins [31]. In G_1 _phase, several genes that encode proteins with diverse functions are expressed, including Rbm14, Syncrip and Zranb2. Rbm14 is implicated as a nuclear receptor coactivator, enhancing the transcription process through other coactivators. Syncrip is important for mRNA processing, translation coupled mRNA turnover and splicing [32]. Zranb2 is a zinc finger protein that is required for alternative splicing. This protein exists as at least two splice variants and may interfere with constitutive 5'-splice site selection [33].

#### PML nuclear bodies

PML nuclear bodies are believed to mediate DNA transcription and DNA damage response [13,14]. During the cell cycle, 7 of 10 PML body genes with peaktime assigned are expressed in S phase. Among those genes, several genes that play an essential role in transcription are expressed in the early stage of this phase, including Runx1 and Zfp51. The precise mechanism of how PML nuclear bodies control transcription is unclear. Zhong et al. report that PML nuclear bodies modulate the availability of transcription factors [34]. Another study suggests that PML nuclear bodies are involved in chromatin remodelling processes [35]. Changes to composition and location of PML nuclear bodies during the cell cycle appear to influence transcription [14].

Cell cycle checkpoint proteins verify that processes at each phase are accurately completed before progressing to the next. In our data we find Topbp1 and Chek2 that respond to cellular stress (to arrest the cell cycle). Proteins encoded by these genes are also putative tumor suppressors. These proteins have a forkhead-associated protein interaction domain that is important for activation in response to DNA damage. In S phase, these proteins also co-localize with Brca1 to restore survival after DNA damage. Topbp1 is also involved in initiation of DNA replication.

Several multi-compartment proteins are coded by genes periodically expressed during the cell cycle. Nono, Cdk9 and Dhx8 localize to nucleoli and nuclear speckles. Topbp1 and Cbx3 localize to both nucleoli and PML nuclear bodies. Nono is DNA and RNA binding protein, and enhances the binding of conventional sequence-specific transcription factors to their recognition sites [36].

## Discussion

The availability of large-scale data of intra-nuclear proteins provides a unique opportunity to explore different features for characterizing individual compartments on basis of their protein complement and for discriminating proteins of one compartment from another. Exploration of several features including gene annotations, amino acid sequence, protein-protein interaction data, and cell cycle expression sheds light on the *functional *organization of *physically *embodied intra-nuclear compartments. Here, we discuss our "data-driven" analyses compartment by compartment, after a few general observations.

Using gene ontology term enrichment analysis, the functional homogeneity of a group of gene products can be explored. For each compartment, we collect the constituent proteins that are identified by recent experimental work, and determine significant GO terms in a nuclear protein context. Similarly, we investigate the enrichment of two sequence features, essential for nuclear import (nuclear localization signals) and nuclear organization (sumoylation sites).

Our analyses also illustrate the relationships between compartments. The fluid exchange of many proteins illustrate a functional relationship. We note that almost all bodies share proteins. Additionally, interactions between proteins (an indicator of functional co-participation) are found both within compartments and between compartments. The large proportion of observed intra-compartment interactions support that protein-protein interaction is a major driver for intra-nuclear organization. Amino acid composition and subtle sequence similarities between proteins of different compartments are also potential indicators of functional similarity. We build classifiers that are able to detect such features. Their *in*ability to distinguish between two compartments illustrates the overlap and sharing of sequence features of proteins associated with those two compartments (see Table 4). In particular, we note that the nucleolar predictor picks up an inproportionate number of PML body and Cajal body proteins (and the Cajal body predictor identifies a fair number of nucleolar proteins). It also seems the PML body predictor is confounded by nuclear speckle proteins and vice versa. We note that several studies suggest that these bodies are indeed physically juxtaposed or transiently linked in some situations [13,26].

### Nucleolus

Broadly, we confirm previous observations that the nucleolus is heavily involved in ribosomal biogenesis. There are 578 known nucleolar proteins in our training set, but the nucleolus classifier predicts that an additional 50%, 287 proteins are missing. Differential expression of nucleolar genes is seen in S and G_2 _stages of the cell cycle. Anecdotal observations of periodically expressed genes identify roles for the nucleolus in stress response and DNA replication.

### Nuclear speckles

In line with previous studies, annotation enrichment analysis of nuclear speckle proteins identify RNA splicing as the core activity of this compartment. It is also significantly enriched in NLS-carrying proteins (38%) relative other nuclear-imported proteins, indicating a relationship with the *classical *nuclear import machinery (based on *a*- and *β*-importins). Using our nuclear speckle classifier, we suggest that there are many more nuclear speckle proteins to be discovered experimentally. Currently, 392 are known but another 308 (87%) are predicted. Periodic expression was only found for 21/392 genes, primarily expressed during the G_1 _and S phases.

### PML nuclear body

Annotations of PML nuclear body proteins show an enrichment of terms relating to cell cycle control, stress response and DNA repair--all in agreement with the literature. The term "Protein modification process" is also identified, lending support to the hypothesis that sumoylation is critical for this nuclear body [1]. The prevalence of sumoylation motifs in member proteins is also higher than in any of the other compartments. On top of the 83 known, about 52 new PML nuclear proteins are predicted but the classification accuracy is poor. 10 of the 83 genes responsible for the PML body proteins are periodically expressed, mostly during the S phase.

### Cajal body

Only 36 proteins are known to associate with Cajal bodies. We find no significant GO terms at *E *< 0.01, but "spliceosome assembly" at *E *< 1. With such a limited data set, building a classifier is premature. We estimate, however, that another 55 Cajal body proteins are still to be discovered (but the false discovery rate is at a discouraging 0.86).

## Conclusions

We expected that the data-driven analyses would demonstrate that intra-nuclear compartments are functionally homogeneous and that their members would exhibit properties that enable their sorting. The collected large-scale protein data provides only partial evidence that the physical organization of the nucleus mirrors functional modularity.

The current data set is far from completely annotated, suggesting that our methods for *evaluating *prediction accuracy are not robust. The error rates may be lower than what we have been able to conclude-ultimately we should instead rely on experimental validation. With the incomplete "trusted" data set, we fail to unambiguously identify proteins' intra-nuclear destination, suggesting that critical drivers behind of intra-nuclear translocation are yet to be identified.

## Methods

### Nuclear localization data set

The data relating to the intra-nuclear localization proteins used in this study are sourced from several databases. As a starting point, we retrieved proteins and their respective intra-nuclear compartments from specialized nuclear proteome databases such as Nuclear Protein Database (NPD) [7] and the Nucleolar Proteome Database (NoPdb) [37]. We added proteins from generic databases such as UniProt KnowledgeBase [8] and Human Protein Reference Database (HPRD) [9] when their location was one of the nominated intra-nuclear compartments. This dataset thus primarily contains human proteins that are experimentally or computationally determined to localize to the nucleus, some with more specific compartments assigned.

Using BioMart and information retrieved from Mouse Genome Informatics database (MGI) [38], the proteins from the data set above are mapped via orthologous genes to mouse protein identifiers. These proteins are then intersected with the full Nucprot mouse nuclear proteome data set [39]. Nucprot identifies 2568 proteins with clear experimental evidence of nuclear localization and 2854 mouse proteins predicted to be nuclear by a number of computational methods. Intra-nuclear association is not provided by Nucprot. The intersection of the compartment-annotated proteins with the Nucprot data set, result in 2448 proteins, all with support of nuclear import from at least two sources.

Proteins assigned with intra-nuclear compartment, but not identified in Nucprot, are subject to a Blast search against Nucprot. If the E-value is smaller than 10^-4^, the Nucprot entry is incorporated in our set, with the designated intra-nuclear compartment. Finally, we extend our set from compartment-specific reviews and large-scale proteomics manuscripts: Andersen et al. (2002) [40], Cioce et al. (2005) [16], Fox et al. (2002) [41] and Cronshaw et al. (2002) [42]. Proteins are included if experimental evidence clearly demonstrates their localization.

The final set consists of 2603 proteins with 1293 proteins with one or more experimentally confirmed intra-nuclear compartments (of which 956 associate with one or more of Nucleoli, Nuclear speckles, PML nuclear bodies and Cajal bodies). The remaining 1310 proteins are only known to localize generally to the nucleus.

### Protein interaction data set

Protein-protein interaction evidence utilized in this study was extracted from HPRD [9]. Similar to the compartment-assigned protein set, each interaction is mapped onto mouse identifiers using BioMart and the MGI [38]. In our analyses, we distinguish between interactions involving proteins that are annotated with a single nuclear body each, and those involving one or two proteins with less than or more than one nuclear body each. The former category can be further divided into intra- and inter-compartment interactions--two sets that unambiguously illustrate the protein-based interaction between compartments. In our analyses, if an interacting protein belongs to less than or more than one nuclear body, we count it as 'Other'.

### Machine learning classification

Support-vector machines are trained to discriminate between positive and negative samples, i.e. to generate a decision function(1)

where *y*_*i*_ϵ{-1,+1} is the target class for sample *i*ϵ{1, ..., *N*}, and *x*_*i *_is the *i*th sample, α_*i *_is the *i *th Lagrange multiplier and *b *is a threshold. All multipliers and the threshold are tuned by training the SVM on 4/5 of the training data, randomly selected. Due to the graded membership of intra-nuclear compartments, the SVM output is converted to a probabilistic output, using a sigmoid function(2)

where *A *and *B *are estimated from the remaining 1/5 of the training data.

A number of sequence-based kernels have been developed recently, primarily targeted to protein classification problems. We evaluate the performance of the Spectrum kernel [43], Wildcard kernel [43], and the Local Alignment kernel [24], replacing *K *in Equation 1.

Essentially, spectrum-based kernels (including the Wildcard kernel) are based on the sharing of short sequence segments (of a specified length, with provision of minor differences in the case of the Wildcard kernel). The Local Alignment kernel compares two sequences by exploring their alignments. An alignment between two sequences is quantified using an amino acid substitution matrix and a gap penalty setting, both of which are set as prescribed in Saigo et al. [24].

Each SVM is trained and tested using 5-fold cross-validation. This procedure is repeated ten times with different data set divisions to establish the variance in classification accuracy.

### Statistical enrichment analysis

The statistical enrichment analyses are all based on the Fisher's Exact Test. That is, we count the number of proteins from the positive class (e.g. for a given compartment) and the negative class (e.g. for all other compartments), and then distinguish between proteins that are assigned a specific property (e.g. have a specified GO term) from those that do not. The null hypothesis is that the positive and negative sets do not differ in terms of their assigned properties. The test establishes a *p*-value, the total probability of observing data as extreme or more extreme, given that the null hypothesis is true.

Notably in the case of GO term analysis, there may be cases where terms are chosen by annotators on basis of experimental evidence of actual compartment association, and the analysis must be interpreted with this in mind. The most obvious example is that "GO:0005730 Cellular component: Nucleolus" is inevitably based on the same evidence as our compartment annotation. We exclude from consideration all cellular component terms that correspond to the compartments in this study and their parts. To reduce the bias-short of removing it-random GO terms are taken from only nuclear imported proteins *with *compartment annotations. (For example, the chance of seeing say "RNA binding" amongst nuclear proteins is much greater than in proteins in general.)

In the case of GO analysis, we list only terms that have a corrected *p*-value (per a Bonferroni correction to determine an *E*-value) of less than 0.01. The Gene Ontology (24 Aug 2007) and *M. musculus *gene annotations (19 Dec 2008) were retrieved from http://www.geneontology.org.

## Authors' contributions

NM and MB jointly wrote the article. NM performed the collection of intra-nuclear proteins data. MB developed the machine learning classifier. NM performed the statistical analysis with the advice of MB. Both authors read and approved the final manuscript.

## Supplementary Material

Additional file 1 **Proteins with observed intra-nuclear compartment(s)**. This file contains a list of all proteins used in this study identified by their UniProtKB accession name. Each protein identifier is followed by zero or more compartments with which the protein is *observed *according to available data sets (see Methods). Click here for file

Additional file 2 **Proteins with predicted intra-nuclear compartment(s)**. Following the same format as Additional file 1, this file lists *predicted *compartments for proteins that have no observed compartment. Click here for file
